# Phytochemical Analysis, Antioxidant, and Wound Healing Activity of *Pluchea indica* L. (Less) Branch Extract Nanoparticles

**DOI:** 10.3390/molecules27030635

**Published:** 2022-01-19

**Authors:** Rattanakorn Chiangnoon, Weerasak Samee, Pimpon Uttayarat, Wullapa Jittachai, Warintorn Ruksiriwanich, Sarana Rose Sommano, Sirivan Athikomkulchai, Chuda Chittasupho

**Affiliations:** 1 Nuclear Technology Research and Development Center, Thailand Institute of Nuclear Technology, Nakhon Nayok 26120, Thailand; rattanakorn@tint.or.th (R.C.); pimponu@tint.or.th (P.U.); 2 Department of Pharmacognosy, Faculty of Pharmacy, Srinakharinwirot University, Nakhon Nayok 26120, Thailand; wullapa@g.swu.ac.th; 3 Department of Pharmaceutical Chemistry, Faculty of Pharmacy, Srinakharinwirot University, Nakhon Nayok 26120, Thailand; weerasak@g.swu.ac.th; 4 Department of Pharmaceutical Sciences, Faculty of Pharmacy, Chiang Mai University, Chiang Mai 50200, Thailand; warintorn.ruksiri@cmu.ac.th; 5 Cluster of Research and Development of Pharmaceutical and Natural Products Innovation for Human or Animal, Chiang Mai University, Chiang Mai 50200, Thailand; sarana.s@cmu.ac.th; 6 Plant Bioactive Compound Laboratory, Department of Plant and Soil Sciences, Faculty of Agriculture, Chiang Mai University, Chiang Mai 50200, Thailand

**Keywords:** HPLC, solvent displacement method, scratch assay, wound healing, cell viability, phytochemical compounds

## Abstract

Proliferation and migration of keratinocytes and fibroblasts play an important role in cutaneous wound healing, while oral mucosal squamous cell proliferation and migration are crucial for oral wound healing. In this study, the phytochemical profile of *Pluchea indica* branch ethanolic extract was characterized. The bioactive compound of *Pluchea indica* branch ethanolic extract was identified and analyzed by the validated HPLC method. The nanoparticles of *P. indica* branch extract were formulated by solvent displacement method to increase the solubility and the colloidal stability of the extract. The stability of the nanoparticles was investigated by using the dynamic light scattering technique. Effects of *P. indica* crude extract and nanoparticles on cell viability, proliferation and migration of primary epidermal keratinocytes, human dermal fibroblasts, and oral mucosal keratinocyte cells were investigated by MTT assay and scratch assay, respectively. The results showed that *P. indica* branch extract contained a high content of total phenolic and total flavonoids. The HPLC analysis revealed that the main compound in the extract was 4,5-*O*-dicaffeoylquinic acid. The cell viability of the extract and nanoparticles decreased when cells were exposed to a high concentration of extract and nanoparticles. These results demonstrate that *P. indica* branch extract and extract nanoparticles at specific concentrations possess in vitro wound healing activity and they may be possibly used to treat different types of wounds including dermal and oral mucosal wounds.

## 1. Introduction

The wound healing process consists of four main phases, including hemostasis, inflammation, proliferation, and remodeling [1]. One of the most important aspects of wound healing is re-epithelialization, which is restoring epithelium by keratinocytes during the proliferative phase. Re-epithelization involves new blood vessel formation, proliferation and migration of cells, such as keratinocytes and fibroblasts [2]. After the skin is injured, diverse cell types are recruited to the wound area. Inflammatory cells are attracted to the infection site and remove the damaged tissues. Keratinocytes and fibroblasts migrate to the wound lesion and proliferate to generate new tissue. In the epithelization stage, the epithelial cells proliferate and migrate from the wound borders to close the wound. The damaged epidermis is regenerated by two main mechanisms involving the activation of epidermal keratinocytes in the wound margin and proliferation of epidermal keratinocytes [3]. Keratinocytes are the predominant cell types of the epidermis and play an important role in the wound healing process. As soon as a monolayer of keratinocytes completely covers the surface of the wound lesion, the epidermal migration is ceased [4,5]. Fibroblasts are the most common cell type in connective tissue. Fibroblasts are the major cell type in the dermis. Fibroblasts play a key role in the deposition of extracellular matrix (ECM) components, wound contraction and remodeling by promoting a change in the composition of the ECM of fibers and cells [6]. Multiple factors can affect wound healing by interfering with one or more of the healing processes, resulting in impaired tissue repair. These can be divided into local factors, such as oxygenation, infection, and vascular insufficiency. The systemic factors include age, stress, ischemia, diabetes, obesity, medication, malnutrition, and immunosuppression [1]. Wound healing impairment generally fails to progress through the normal healing process and causes some consequences, including pathologic inflammation and chronic wounds. The oral mucosa has faster wound healing with minimal scar formation than skin wound due to its more moistened environment in the oral civility [7,8]. The process of oral mucosa wound healing is similar to the skin. In this study, we used a buccal mucosa squamous cell to investigate the effect of *P. indica* branch extract and NPs on oral mucosal wound healing.

*Pluchea indica* L. (Less) is a plant species in the Asteraceae family. Several reports show the wound healing property of crude extract of different parts of *P. indica*. We previously reported that *P. indica* leaf extract could enhance the migration of oral mucosa cells, indicating the ethanol extract’s wound healing property of the ethanol extract of *P. indica* leaf [9]. Pramanik et al. reported that methanol root extract of *P. indica* revealed wound healing activity significantly in an animal model by enhancing the rate of wound contraction, decreasing epithelization period increasing skin breaking strength, increasing dry granulation tissue weight, and increasing collagen formation [10]. *P. indica* root, stem, and twig extracts showed high antioxidant and antibacterial activities. The antioxidant capacities of *P. indica* (L.) Less extracts might be due to the electron or hydrogen donating ability of phenolic compounds [11]. However, to the best of our knowledge, *P. indica* branch extract has not been investigated for its antioxidant and wound healing properties. In addition, the major bioactive compound in the branch extract has not been previously studied.

The topical route of administration has limitations from the low bioavailability due to poor drug penetration through the skin. The advantages of using nanocarrier as a drug delivery system include its ability to improve skin penetration of bioactive molecules, increase drug deposition in the target region, enhance drug physical and chemical stability, and control the release of the active compounds [12]. Nematallah et al. demonstrated that SMEDDS loading polyphenols-enriched fraction of Ajwa fruit enhanced oral bioavailability of the fraction and hepatoprotective effect [13]. Previous reports have shown that nanoparticles prepared by solvent displacement method have been shown to enhance water solubility and stability of capsaicin and lutein [14,15].

In this study, the bioactive compound of *P. indica* branch extract was identified with the validated HPLC method. The effects of *P. indica* branch ethanol extract on the proliferation and migration of primary epidermal keratinocytes, human dermal fibroblasts, and oral mucosal keratinocyte cells were demonstrated. Future development in bioactive wound healing herbal products based on *P. indica* branch extract is indicated using the increased information of the action of this extract.

## 2. Materials and Methods

### 2.1. Materials

Branches of *P. indica* were collected from the herbal garden of the Faculty of Pharmacy, Srinakharinwirot University, in August 2018 and was identified by Assistant Professor Dr. Sirivan Athikomkulchai. The plant specimen was deposited (SIRA002) at the herbarium of the Faculty of Pharmacy, Srinakharinwirot University. Primary Epidermal keratinocytes (ATCC^®^ Number: PCS-200-011), human dermal fibroblast cells line (HDFB) and dermal cell basal medium plus keratinocyte growth kit were purchased from the American Type Culture Collection (Manassas, VA, USA). Dulbecco’s modified eagle’s medium (DMEM), fetal bovine serum (FBS), trypsin-EDTA, sodium carboxymethyl cellulose, 3-(4,5-Dimethylthiazol-2-yl)-2,5-Diphenyltetrazolium Bromide (MTT), Epigallocatechin gallate (EGCG) standard, gallic acid standard, sodium nitrite, and sodium hydroxide, and dimethyl sulfoxide (cell culture grade) were purchased from Sigma Aldrich (St. Louis, MO, USA). Folin-Ciocaleu reagent, 95% ethanol, sodium carbonate, and aluminium chloride were purchased from Merck & Co., Inc. (Kenilworth, NJ, USA). Penicillin-streptomycin and phosphate-buffered saline were purchased from Gibco Thermo Fisher Scientific (Waltham, MA, USA). Phosphoric acid and acetonitrile were purchased from RCI Labscan, Bangkok, Thailand.

### 2.2. Preparation of P. indica Branch Extract

The fresh branches of *P. indica* were washed, air-dried followed by hot air oven-dried at 50 °C for 3 h. The dried branches were coarsely powdered. For extraction by refluxing, 100 g of powdered plant material was put in the thimble, mixed with 500 mL of 95% ethanol in a round bottom flask, and refluxed for 3 h using a soxhlet extractor. The solvent was removed by using a rotary evaporator. The yield (%) of extraction was calculated by Equation (1):

(1) $$\mathrm{Yield}\;\mathrm{of}\;\mathrm{extraction}\;\left(\%\right)=\frac{\mathrm{Weight}\;\mathrm{of}\;\mathrm{crude}\;\mathrm{extract}}{\mathrm{Weight}\;\mathrm{of}\;\mathrm{powdered}\;\mathrm{branches}}\times 100$$

### 2.3. Phytochemical Screening of P. indica Branch Extract

#### 2.3.1. Test for Flavonoids

The presence of flavonoids in *P. indica* branch extract was determined by the Shinoda test. Crude extract of *P. indica* branch was dissolved in 95% ethanol at a concentration of 1000 µg/mL and was mixed with five fragments of magnesium ribbons. The 37% hydrochloric acid (1 mL) was added dropwise. The formation of a red-pink color indicated the presence of flavonoids in the extract.

#### 2.3.2. Test for Phenolic Compounds

Phenolic compounds in the extract were identified by ferric chloride test. The crude extract of *P. indica* branch in 95% ethanol at 1000 µg/mL was mixed with 1 mL of 2.5% ferric chloride solution. The formation of a greenish-blue color indicated the presence of phenolic compounds.

#### 2.3.3. Test for Tannins

The following solution was added into the crude extract of *P. indica* branch in 95% ethanol at 1000 µg/mL and mixed, i.e., 2% *w*/*v* gelatin solution, lead acetate saturated solution, and 1% ferric chloride solution. The precipitation of tannin indicated the presence of tannins.

#### 2.3.4. Test for Alkaloids

The presence of alkaloids in *P. indica* branch extract was detected using Dragendorff’s reagent and Scheibler’s reagent. The 1% crude extract of the *P. indica* branch was diluted with 0.1 N HCl. Dragendorff’s reagent or Scheibler’s reagent containing potassium bismuth iodide and phosphotungstic acid, respectively, was added into the extract suspension. The orange and white precipitations in Dragendorff’s test or Scheibler’s test, respectively, were observed for positive results of alkaloids.

#### 2.3.5. Test for Terpenoids

Salkowski test was used to detect terpenoids. The crude extract of the *P. indica* branch (0.5 g) was mixed with dichloromethane (2 mL), and 98% sulfuric acid (3 mL) was carefully added to form a layer. The presence of terpenoids in the extract was shown by the reddish-brown color ring on the sulfuric acid and dichloromethane interface.

### 2.4. Determination of Total Phenolic Content in P. indica Branch Extract

Total phenolic content in the crude extract of the *P. indica* branch was determined by the Folin–Ciocalteu method described by [14]. Gallic acid was dissolved in 95% ethanol at 10 mg/mL as a stock solution. The standard curve of gallic acid was prepared by dilution of gallic acid stock solution with deionized water to give 50, 100, 150, 200, 250, 300, 350, 400, 450, and 500 µg/mL. The crude extract of the *P. indica* branch was dissolved in 95% ethanol at 3, 5, and 10 mg/mL. The crude extract or gallic acid solution (40 µL) was mixed with Folin–Ciocalteu reagent (40 µL). Then, 7.5% Na_2_CO_3_ (800 µL) and deionized water (1120 µL) were added. The mixture was incubated at room temperature for 60 min. The mixture was then transferred to a 96-well plate (200 µL/well). The absorbance of the extract and the standard solutions was measured against the reagent blank at 765 nm using a UV-Vis spectrophotometer microplate reader (Spectramax M3, Thermo Fisher Scientific, Waltham, MA, USA). The total phenolic content in the extract was determined from the calibration curve and expressed as micrograms of gallic acid equivalent (µg GAE) per milligram of crude extract. The experiment was repeated thrice for all concentrations of the extracts and standards.

### 2.5. Determination of Total Flavonoid Content in P. indica Branch Extract

Total flavonoid content in *P. indica* branch extract was determined using aluminum chloride colorimetry assay. EGCG was dissolved in 95% ethanol at 5 mg/mL as a stock solution and was diluted with 50% ethanol to give standard solutions at concentrations of 23, 34.5, 46, 57.5, 69, 100, and 250 µg/mL. The crude extract was dissolved in 95% ethanol at 3, 5, and 10 mg/mL. The crude extract or standard EGCG solution was mixed with 5% NaNO_2_ (300 µL). The mixture was incubated at room temperature for 5 min. Then, 2% aluminium chloride (AlCl_3_) solution (500 µL) was added to the mixture. The mixture was further incubated for 6 min followed by adding 1 N sodium hydroxide (500 µL). The mixture was vigorously mixed and incubated at room temperature for 10 min. The solution was pipetted to a 96-well plate (200 µL/well), and the absorbance was measured at 510 nm using a UV/Vis spectrophotometer microplate reader (Spectramax, Thermo Fisher Scientific, Waltham, MA, USA). The total flavonoid content was determined from the calibration curve and expressed as micrograms of EGCG equivalent per milligram of crude extract (µg CE/mg Extract). The experiment was repeated thrice for all concentrations of the extracts and standards.

### 2.6. Determination of Bioactive Compound in P. indica Branch Extract by High-Performance Liquid Chromatography (HPLC)

#### 2.6.1. Equipment and Chromatographic Conditions

HPLC separation was achieved on the YL9100 Plus HPLC (Young in Chromass, Korea) equipped with the YL9111 binary pump, YL9150 autosampler, YL9131 column compartment, and YL9120 UV/VIS detector. The separation was performed in an ACE 5 C_18_-AR column (4.6 × 250 mm, 5 µm) with a C_18_ guard column. The mobile phases were (A) 0.085% phosphoric acid in water and (B) acetonitrile using gradient elution: 10% B in A to 40% B in A for 40 min; 95% B for 10 min prior for each analysis, and the flow rate was set at 1.0 mL/min with the controlled temperature at 25 °C. The UV detector was set at the wavelength of 326 nm and the injection volume was 20 μL for every sample and reference standard.

#### 2.6.2. Sample Preparation

Each sample was prepared by accurately weighing *P. indica* branch extract and dissolving in the mobile phase (50% of 0.085% phosphoric acid in water and 50% of ACN) (2000 µg/mL). Prior to injection, each sample was filtered through a 0.45 µm nylon membrane and then analyzed in triplicate.

#### 2.6.3. Stock and Working Solution of Standards

The stock solution of the 4,5-*O*-dicaffeoylquinic acid standard was prepared by accurately weighing and dissolving the standard in the mobile phase to obtain the final concentration of 200 µg/mL. A working solution of standard compounds was obtained by diluting the standard stock solutions with mobile phase to achieve the desired concentrations.

### 2.7. Method Validation of HPLC Analysis

The HPLC method was validated for linearity, precision, accuracy, limit of detection (LOD) and limit of quantitation (LOQ) according to the International Conference on Harmonization guidelines (ICH, 1996/2005) and the acceptable range according to AOAC 2002 guideline.

#### 2.7.1. Linearity

Linearity was determined by using working standard solutions of 4,5-*O*-dicaffeoylquinic acid at concentrations of 10, 8, 6, 4, and 2 µg/mL. Each concentration was analyzed in triplicate. The calibration curves were obtained by plotting the peak area versus the concentration of 4,5-*O*-dicaffeoylquinic acid standard.

#### 2.7.2. Precision

The intraday precision was determined by analyzing standard solutions of 4,5-*O*-dicaffeoylquinic acid at concentrations of 9, 6 and 3 µg/mL three times within one day. The interday precision was examined for three consecutive days by the proposed method. The precision was expressed as percent relative standard deviation (%RSD).

#### 2.7.3. Accuracy

Recovery was used to evaluate the accuracy of the method. The standard solutions of 4,5-*O*-dicaffeoylquinic acid at concentrations of 9, 6 and 3 µg/mL were analyzed. The recovery was calculated by the following equation (Equation (2)):

(2) $$\mathrm{Recovery}\;\left(\%\right)=\frac{\left(\mathrm{Amount}\;\mathrm{found}-\mathrm{Amount}\;\mathrm{added}\right)}{\mathrm{Amount}\;\mathrm{added}}\times 100\%$$

#### 2.7.4. Limit of Detection (LOD) and Limit of Quantitation (LOQ)

LOD and LOQ were achieved by calculating from three calibration curves by the following equations.

(3) $$\mathrm{LOD}=\frac{3\mathrm{SD}\;\mathrm{of}\;\mathrm{intercepts}}{\mathrm{Mean}\;\mathrm{of}\;\mathrm{slope}}$$

(4) $$\mathrm{LOQ}=\frac{10\mathrm{SD}\;\mathrm{of}\;\mathrm{intercepts}}{\mathrm{Mean}\;\mathrm{of}\;\mathrm{slope}}$$

### 2.8. Preparation of P. indica Branch Extract Nanoparticles

The nanoparticles of *P. indica* branch extract were prepared by solvent displacement method [16,17]. *P. indica* branch extract (30 mg) was dissolved in 95% ethanol (2 mL). The extract solution was infused into 0.1% sodium carboxymethyl cellulose or 0.1% poloxamer 407 (15 mL) at a rate of 10 mL/h, under magnetic stirring (540 rpm). The extract NPs were dialyzed against deionized water for 12 h to remove the residual ethanol.

### 2.9. Characterization and Stability Study of P. indica Branch Extract NPs

The hydrodynamic diameter, polydispersity index, and zeta potential values of *P. indica* branch extract dispersed in deionized water and freshly prepared *P. indica* branch extract NPs were determined by the dynamic light scattering technique and electrophoretic mobility using a Zetasizer Nano ZS (Malvern Instruments, Malvern, UK). The stability of the extract NPs was investigated by storage of the NPs at 4 °C for 4 weeks. At 1, 2, 3, and 4 weeks, the size, size distribution, and the surface charge of the NPs were measured.

### 2.10. Determination of DPPH Free Radical Scavenging Activity of P. indica Branch Extract and NPs

The DPPH free radical scavenging capacity of the *P. indica* branch extract and NPs was determined and compared with gallic acid. Gallic acid was diluted in deionized water at concentrations of 3.9–2000 µg/mL. *P. indica* branch extract and NPs were diluted in deionized water at concentrations of 0.2–50 mg/mL and 0.03–6.67 mg/mL, respectively. DPPH was dissolved in absolute ethanol at a concentration of 500 µM. Gallic acid, *P. indica* branch extract, and NPs (100 µL) were mixed with DPPH solution (100 µL). The mixture was incubated at room temperature in the dark for 30 min. The absorbance was measured using a UV-Vis spectrophotometer microplate reader at a maximum wavelength of 517 nm. The percentage of radical scavenging activity was calculated by Equation (5). The 50% of scavenging (IC50) was calculated from the non-linear regression analysis of the graph plotted between the percentages of DPPH free radical scavenging and the sample concentrations.

(5) $$\mathrm{DPPH}\;\mathrm{free}\;\mathrm{radical}\;\mathrm{scavenging}\;\left(\%\right)\frac{\left(A-B\right)}{A}\times 100$$

where *A* was the absorbance of the reaction with solvent control and *B* was the absorbance of reaction with the extract.

### 2.11. Determination of ABTS Free Radical Scavenging Activity of P. indica Branch Extract and NPs

Gallic acid was diluted in deionized water at concentrations of 3.9–2000 µg/mL. *P. indica* branch extract and NPs were diluted in deionized water at concentrations of 0.2–50 mg/mL and 0.03–6.67 mg/mL, respectively. ABTS was dissolved in absolute ethanol to 7 mM concentration. ABTS radical cation (ABTS+) was produced by reacting ABTS stock solution with 2.45 mM potassium persulfate was dissolved in and kept in the dark at room temperature for 24 h before use. The ABTS+ solution was diluted with absolute ethanol to an absorbance of 0.700 (±0.02) at 734 nm. Gallic acid, *P. indica* branch extract and NPs (180 µL) were mixed with ABTS solution (20 µL). The mixture was incubated at room temperature in the dark for 15 min. The absorbance was measured using a UV-Vis spectrophotometer microplate reader at a wavelength of 734 nm. The percentage of radical scavenging activity was calculated by Equation (6). The 50% of scavenging (IC_50_) was calculated from the non-linear regression analysis of the graph plotted between the percentages of ABTS+ free radical scavenging and the sample concentrations.

(6) $$\mathrm{ABTS}\;\mathrm{free}\;\mathrm{radical}\;\mathrm{scavenging}\;\left(\%\right)\frac{\left(A-B\right)}{A}\times 100$$

where *A* was the absorbance of the reaction with solvent control and *B* was the absorbance of reaction with the extract.

### 2.12. Determination of Ferric Reducing Antioxidant Power (FRAP) of P. indica Branch Extract and NPs

Gallic acid was diluted in deionized water at concentrations of 3.9–2000 µg/mL. *P. indica* branch extract and NPs were diluted in deionized water at concentrations of 0.1–50 mg/mL and 0.03–6.67 mg/mL, respectively. FRAP reagent was prepared from acetate buffer (300 mM, pH 3.6), a solution of 10 mM TPTZ in 40 mM HCl, and 20 mM FeCl3 at 10:1:1 (*v*/*v*). The FRAP reagent (180 µL) and sample solutions (20 µL) were added to each well and mixed thoroughly. The mixture was incubated at 37 °C for 30 min. Then, the absorbance was read at 595 nm. The standard curve was constructed using ferrous sulfate solution (9.8–5000 μM), and the results were expressed as μmol Fe (II) equivalent.

### 2.13. Cell Culture

Primary epidermal keratinocytes were cultured in dermal cell basal medium plus keratinocyte growth kit supplemented with 1% penicillin-streptomycin and maintained at 37 °C in a humidified incubator containing an atmosphere of 5% CO_2_. Human dermal fibroblasts (HDFB) were cultured in Dulbecco’s modified eagle’ medium (DMEM) supplemented with 10% fetal bovine serum and 1% penicillin-streptomycin and maintained at 37 °C in a humidified incubator containing an atmosphere of 5% CO_2_. HO-1-N-1 cells were cultured in DMEM-F12 (1:1) medium supplemented with 10% fetal bovine serum and 1% penicillin-streptomycin and maintained at 37 °C in a humidified incubator containing an atmosphere of 5% CO_2_.

### 2.14. The Viability Study of Primary Epidermal Keratinocytes, Human Dermal Fibroblast (HDFB) and HO-1-N-1 Cell Lines

Primary epidermal keratinocytes (1 × 10^5^ cells/well), human dermal fibroblast (8000 cells/well), and HO-1-N-1 cells (1.5 × 10^3^ cells/well) were plated in 96-well plates and incubated for 24 h at 37 °C, 5% CO_2_ before the test. The medium was removed, and the extract and NPs suspended in a 2% fetal bovine serum-containing medium (200 µL) at various concentrations. Primary epidermal keratinocytes were incubated with 62.5, 125, 250, 500 and 1000 µg/mL extract and NPs. Human dermal fibroblasts were incubated with 31.25, 62.5, 125, 250, 500 and 1000 µg/mL of extract and NPs. HO-1-N-1 were incubated with 2.0, 3.9, 7.8, 15.6, 31.3, 62.5, 125, 250, and 500 µg/mL of extract and NPs. The extract and NPs were added to the cells and incubated at 37 °C in 5% CO_2_ for 2 h. After incubation, the extract and NPs were removed, and the cells were washed with PBS. MTT (1 mg/mL) was added to the cells (50 µL/well) and incubated for 3 h at 37 °C in 5% CO_2_. The MTT solution was removed, and 100 μL of DMSO was added to solubilize the water-insoluble formazan product. The absorbance was measured at 570 nm. The cell viability percentage was calculated using Equation (2), where the control was the viability of untreated cells. The IC_50_ was calculated based on the non-linear regression analysis.

(7) $$\mathrm{Cell}\;\mathrm{viability}\;\left(\%\right)=\frac{A570\;\mathrm{of}\;\mathrm{tested}\;\mathrm{cells}}{A570\;\mathrm{of}\;\mathrm{control}}\times 100\%$$

### 2.15. In Vitro Scratch Assay

The effects of *P. indica* branch extract and NPs on the migration of primary epidermal keratinocytes, human dermal fibroblasts, and HO-1-N-1 cells were investigated by the scratch assay to mimic cell migration on the process of wound healing in vivo. Primary epidermal keratinocytes (50,000 cells/well), human dermal fibroblasts (50,000 cells/well), and HO-1-N-1 cells (45,000 cells/well) were cultured in a 24-well plate (at 37 °C, 5% CO_2_ for 24 h. The 100% cell confluence was observed before the scratch assay was performed. A sterile 200 μL pipette tip was used to scratch on the monolayer of cells to simulate a wound. The medium with cell debris was removed. *P. indica* branch extract in 2% fetal bovine serum-containing medium at concentrations of 62.5 and 125 μg/mL were added into the wells and incubated for 2 h at 37 °C, 5% CO_2_. For negative control, cells were not treated and incubated in 2% fetal bovine serum-containing medium. After incubation, the cells were rinsed with 2% fetal bovine serum-containing medium three times and incubated with 2% fetal bovine serum-containing medium. The images of cells were captured under an inverted microscope equipped with a camera (TS100 Nikon, Tokyo, Japan) after 2, 4, 8, and 24 h for primary epidermal keratinocytes, 6, 24, and 48 h for human dermal fibroblasts, and 24, 48, and 72 h for HO-1-N-1 cells. The areas of the wound were analyzed by using ImageJ software. The % wound closure of cells treated with the extract, NPs and control was calculated according to the following Equation (8).

(8) $$\mathrm{Wound}\;\mathrm{closure}\;\left(\%\right)=\frac{\mathrm{Area}\;\mathrm{between}\;\mathrm{cells}\;\mathrm{at}\;0\;h-\mathrm{Area}\;\mathrm{between}\;\mathrm{cells}\;\mathrm{at}\;\mathrm{specified}\;\mathrm{time}}{\mathrm{Area}\;\mathrm{between}\;\mathrm{cells}\;\mathrm{at}\;0\;h}\times 100\%$$

### 2.16. Statistical Analysis

Statistical analysis of data was completed using an analysis of variance (one-way ANOVA), followed by Newman–Keuls method as a posthoc test to evaluate the significance of differences (GraphPad Prism 7.02, La Jolla, CA, USA). In all cases, values of *p* < 0.05, *p* < 0.01, *p* < 0.001, and *p* < 0.0001 were considered statistically significant. 

## 3. Results

### 3.1. Extraction of P. indica Branch Extract

*P. indica* branch extract obtained from maceration with 95% ethanol was concentrated and dried under reduced pressure using a rotary evaporator. The dried extract was weighed, and the yield was calculated to be 7.60 ± 0.56% *w*/*w*.

### 3.2. Phytochemical Compounds in P. indica Branch Extract

Phytochemical constituents including flavonoids, phenolic compounds, tannins, and alkaloids were determined in *P. indica* branch extract. Phytochemical analysis revealed the presence of flavonoids from the Shinoda test, phenolic compounds from the ferric chloride test, tannins from gelatin solution, lead acetate saturated solution, and 1% ferric chloride solution tests, alkaloids from Dragendorff’s and Scheibler’s tests, and terpenoids from Salkoski test. 

### 3.3. Total Phenolic Content in P. indica Branch Extract

Phenolic compounds are critical for antioxidant activity. The hydroxyl groups in the plant extract are responsible for free radical scavenging. The total phenolic content in *P. indica* branch extract was calculated from a calibration curve of gallic acid (y = 0.0011x − 0.039, R^2^ = 0.9981), and the total phenolic content was reported as gallic acid equivalents (GAE) per g of crude extract. The content of phenolic compounds in ethanol extract was 68.37 ± 10.61 mg GAE per 1 g of *P. indica* branch extract. This result suggested that there was 6.83 ± 3.52% *w*/*w* of phenolic compounds in *P. indica* branch crude extract.

### 3.4. Total Flavonoid Content in P. indica Branch Extract

The total flavonoid content of ethanol extract of *P. indica* branch was calculated from a calibration curve of gallic acid (y = 0.0034x − 0.0201, R^2^ = 0.998). The total flavonoid content was expressed as mg EGCG equivalents/g of crude extract. The result revealed that ethanol extract of *P. indica* branch had 11.71 ± 0.03 mg EGCG per 1 g of *P. indica* branch crude extract, which accounted for 1.17 ± 0.03% *w*/*w* of phenolic compounds in *P. indica* branch crude extract.

### 3.5. Determination of Bioactive Compound in P. indica Branch Crude Extract

**Figure 1:**
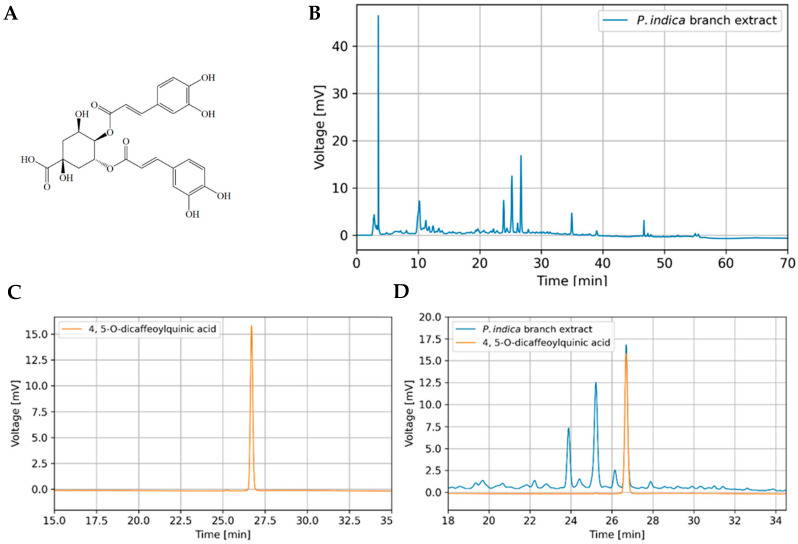
(**A**) Chemical structure of 4,5-*O*-dicaffeoylquinic acid (**B**) HPLC chromatograms of *P. indica* branch extract (**C**) 4,5-*O*-dicaffeoylquinic acid standard and (**D**) overlaid HPLC chromatogram of *P. indica* branch extract and 4,5-*O*-dicaffeoylquinic acid standard.

A validated HPLC method was used for analyzing the contents of 4,5-*O*-dicaffeoylquinic acid (Figure 1A) in *P. indica* branch extract. The HPLC system yielded symmetric peaks and provided the efficient separation of 4,5-*O*-dicaffeoylquinic acid from other compounds in the extract (Figure 1B). The maximal wavelength used for detecting 4,5-*O*-dicaffeoylquinic acid was 326 nm. The chromatograms of standard 4,5-*O*-dicaffeoylquinic acid and overlay of *P. indica* branch extract and 4,5-*O*-dicaffeoylquinic acid are shown in Figure 1C,D, respectively. The retention time of 4,5-*O*-dicaffeoylquinic acid was 26.7 min, and there was no interference in the analysis.

### 3.6. Method Validation of HPLC Analysis

To ensure that the method is suitable for its intended use, the method validation has been performed according to the ICH guidelines (ICH, 1996/2005). The proposed HPLC method showed acceptable validation parameters. The calibration curves were obtained by plotting the peak area versus the concentration of the standards and proved that the method was linear within the range of 2–10 µg/mL for 4,5-*O*-dicaffeoylquinic acid with a coefficient of determination (r^2^) ≥ 0.9990. The percent relative standard deviation (%RSD) of 4,5-*O*-dicaffeoylquinic acid ranged from 0.06–0.89% and 0.50–1.82% for intraday and interday precisions, respectively. The RSD values of intraday and interday precision were lower than 2.0%, reflecting the precision of the assay method. The LOD and LOQ were found to be 0.05 and 0.16 µg/mL, respectively, indicating the high sensitivity of the method. The average recovery percentages of 4,5-*O*-dicaffeoylquinic acid at 3, 6, and 9 µg/mL were 107.51 ± 1.39, 100.18 ± 0.77, and 97.01 ± 0.71, respectively, indicating good accuracy of the analytical method. The average concentration of 4,5-*O*-dicaffeoylquinic acid in the extracts determined in three days was 0.027% *w*/*w*.

### 3.7. Characterization and Stability of P. indica Branch Extract NPs

**Figure 2:**
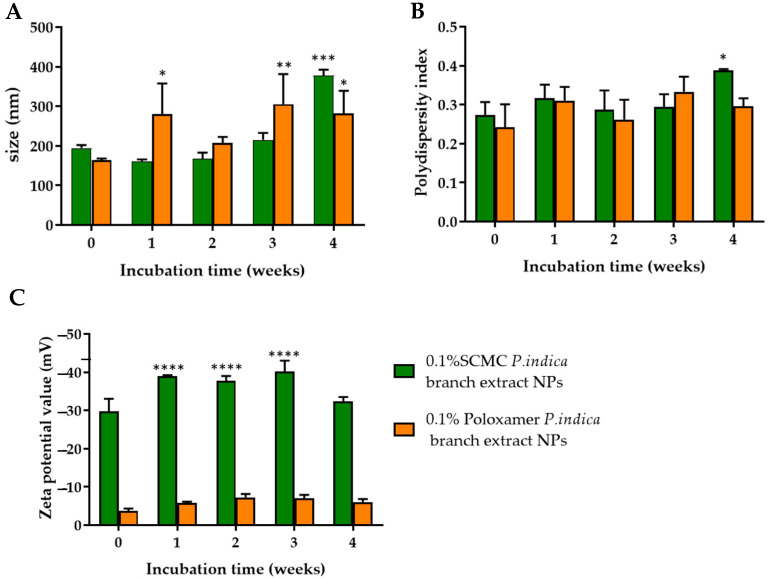
(**A**) Hydrodynamic diameter (**B**) polydispersity index and (**C**) zeta potential values of *P. indica* branch extract NPs after fresh preparation and storage in deionized water for 1, 2, 3, and 4 weeks at 4 °C. The data represent mean ± SD of three experiments. *, **, ***, and **** in dicate *p* < 0.05, *p* < 0.01, *p* < 0.001, and *p* < 0.0001 compared with day 0, respectively.

*P. indica* branch extract dispersed in deionized water had size, polydispersity index, and zeta potential values of 8748.67 ± 3120.02 nm, 0.461 ± 0.486, and −22.77 ± 1.07 mV, respectively. *P. indica* branch extract NPs prepared using 0.1% SCMC and 0.1% poloxamer 407 as stabilizers had an average diameter of 194.67 ± 7.59 nm and 163.73 ± 4.41 nm, respectively. The sizes of the NPs prepared by using 0.1% SCMC stored at 4 °C for 1, 2, and 3 weeks were not significantly altered. The NPs had significantly larger size after storage for 4 weeks, while the NPs prepared using 0.1% poloxamer 407 had significantly increased hydrodynamic diameter after storage for 1, 3, and 4 weeks compared with freshly prepared NPs (Figure 2A). The polydispersity index values of *P. indica* extract NPs prepared using 0.1% SCMC and 0.1% poloxamer 407 at day 0 were 0.27 ± 0.03 and 0.24 ± 0.06, respectively. The polydispersity indexes of the extract NPs did not significantly change after storage for 3 weeks, but at week 4 the PDI was significantly increased (Figure 2B). The polydispersity index values of NPs prepared from 0.1% poloxamer were not changed during storage. The zeta potential values of *P. indica* branch extract NPs prepared from 0.1% SCMC and 0.1% poloxamer were −29.83 ± 3.23 and −1.15 ± 0.06 mV. After incubation for 1, 2, and 3 weeks, the charge of the NPs prepared from 0.1% SCMC became more negative (Figure 2C). However, they were still in the range of −30 to −40 mV, while NPs prepared from 0.1% poloxamer did not change.

### 3.8. DPPH and ABTS Free Radical Scavenging Activities of P. indica Branch Extract and NPs 

**Figure 3:**
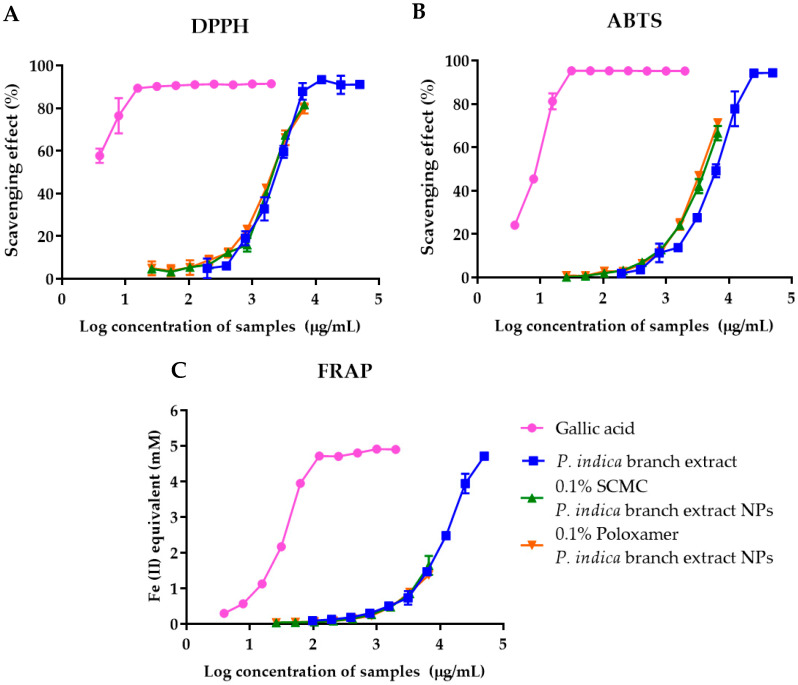
The antioxidant activity of gallic acid, *P. indica* branch extract, 0.1% SCMC *P. indica* branch extract NPs, and 0.1% poloxamer *P. indica* branch extract NPs determined by (**A**) DPPH free radical scavenging assay (**B**) ABTS free radical scavenging assay and (**C**) FRAP assay. The data represent mean ± SD of three experiments.

The radical scavenging activities of *P. indica* branch extract, 0.1% SCMC *P. indica* extract NPs and 0.1% poloxamer *P. indica* extract NPs were compared with gallic acid at various concentrations and expressed as % radical scavenging effect (%) against DPPH and ABTS. The results showed that the radical scavenging activities of the extract and NPs increased with the concentration of the extract and NPs (Figure 3A, B). *P. indica* branch extract, 0.1% SCMC *P. indica* extract NPs, and 0.1% poloxamer *P. indica* extract NPs showed DPPH radical scavenging activity with the IC_50_ values of 2305, 2176, and 2066 µg/mL and ABTS radical scavenging activity with the IC_50_ values of 5769, 4066, 3592 µg/mL. Gallic acid at 3.9 µg/mL presented more than 50% DPPH free radical scavenging activity and had an IC_50_ value of 7.82 µg/mL for ABTS radical scavenging activity. DPPH free radical scavenging activity of *P. indica* extract NPs was comparable with that of the extract, indicating that formulating *P. indica* branch extract in nanoparticles did not affect the free radical scavenging activity of the extract. However, the IC_50_ values of 0.1% SCMC *P. indica* extract NPs and 0.1% poloxamer *P. indica* extract NPs were less than that of extract, indicating the higher ABTS radical scavenging activity of the NPs than the extract; 0.1% SCMC and 0.1% poloxamer did not exhibit DPPH and ABTS free radical scavenging activities. In general, the radical scavenging properties of extract and NPs depend on the chemical composition and the total phenolic compound and flavonoid contents.

### 3.9. Ferric Reducing Antioxidant Potential (FRAP) of P. indica Branch Extract and NPs 

The FRAP assay was performed to determine the reducing capacity of *P. indica* branch extract and NPs in a redox reaction. The standard curve of ferrous sulfate revealed good linearity within the range of 0.01–5 mM (r^2^ = 0.9979). The results of the FRAP assay were expressed as Fe^2+^ equivalent. The ferric reducing power of gallic acid, *P. indica* branch extract, and NPs was dose-dependent (Figure 3C). The 0.1% SCMC and 0.1% poloxamer showed slightly ferric reducing power with 0.04 and 0.05 mM Fe^2+^ equivalent. These results indicated that compared to other mechanisms, the antioxidant activity of *P. indica* branch extract and NPs was partly based on the reducing power of the compounds in the extract, which reduced ferric ions (Fe^3+^) to the ferrous ion.

### 3.10. Effects of P. indica Branch Extract and NPs on Cell Viability of Primary Epidermal Keratinocyte, Human Dermal Fibroblasts, and HO-1-N-1 Cells

**Figure 4:**
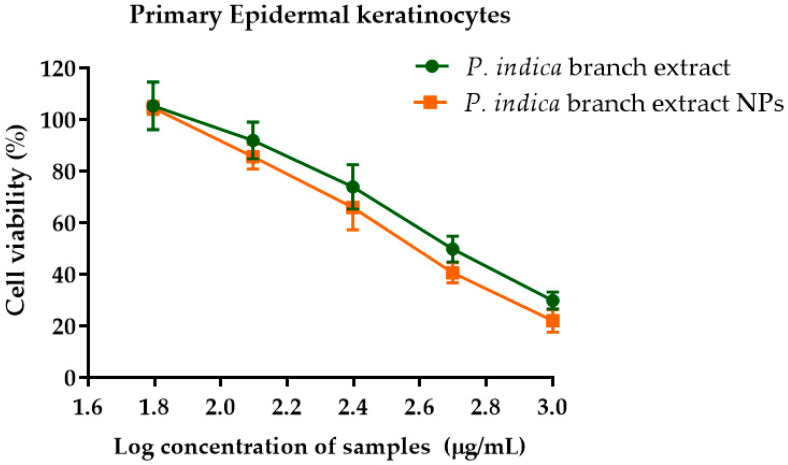
Effect of *P. indica* branch extract and NPs on primary epidermal keratinocyte cell viability after incubation for 24 h. The data represent mean ± SD of three experiments.

**Figure 5:**
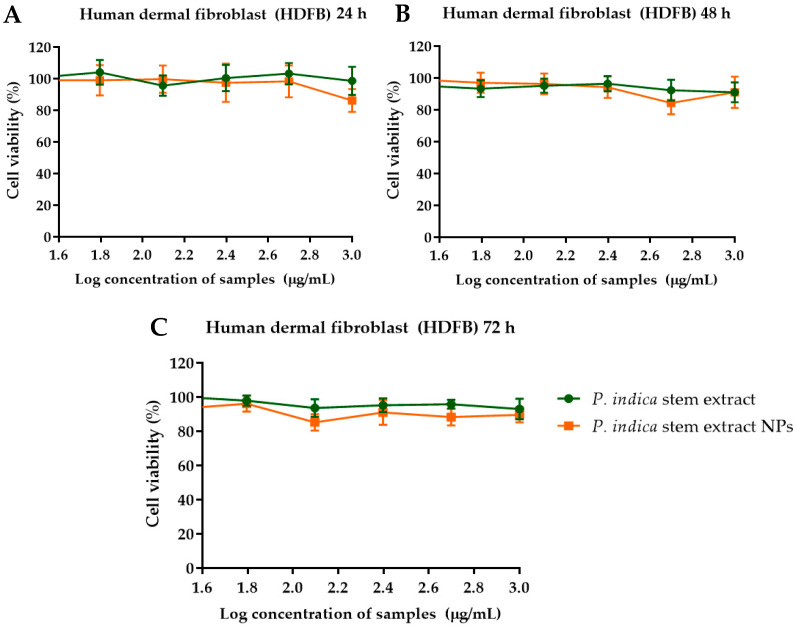
Effect of *P. indica* branch extract and NPs on human dermal fibroblast cell viability after incubation for (**A**) 24 (**B**) 48 and (**C**) 72 h. The data represent mean ± SD of three experiments.

**Figure 6:**
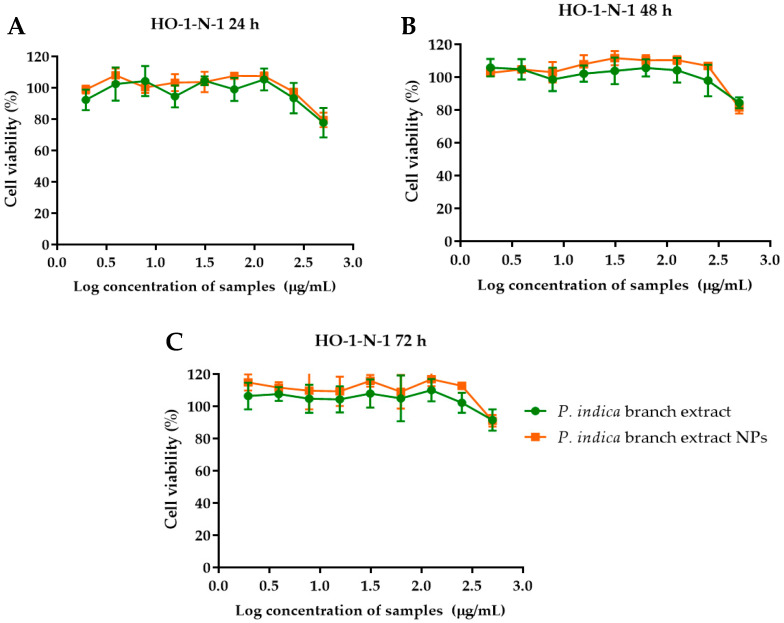
Effect of *P. indica* branch extract and NPs on HO-1-N-1 cell viability after incubation for (**A**) 24 (**B**) 48 and (**C**) 72 h. The data represent mean ± SD of three experiments.

The effects of *P. indica* branch extract and the NPs on primary epidermal keratinocytes, human dermal fibroblasts, and HO-1-N-1 cell lines were investigated using MTT assay. The results showed that primary epidermal keratinocytes were metabolically less active at higher concentrations of *P. indica* branch extract and NPs (Figure 4). The IC_50_ of *P. indica* branch extract and NPs were 453.6 ± 6.9 and 342.2 ± 27.7 µg/mL, respectively (Table 1). In contrast, increased concentrations of *P. indica* branch extract and NPs did not affect the metabolic process of human dermal fibroblasts (Figure 5). At 24h after incubation with the extract and NPs, % cell viability was 95–104%, and 86–99%, respectively. After incubation for 48 h, fibroblast cells exposed to the extract and NPs were 91–96% and 84–99%, respectively. The % viability after exposure to the extract and NPs for 72 h were 93–100% and 88–96%, respectively. These results indicated the biocompatibility of phytochemical constituents of the extract to fibroblast cells. HO-1-N-1 cell metabolic activity was affected when the cells were exposed to 500 µg/mL of the extract and NPs for 24 h. However, at 48 and 72 h, the % cell viability was more than 80%, indicating the biocompatibility of the extract and NPs against the oral mucosal cell line (Figure 6). The results of the MTT assay were used to identify the optimal concentration of the extract and the NPs that can influence the cell viability of keratinocytes, fibroblasts, and oral mucosal keratinocyte cells. 

### 3.11. Effects of P. indica Branch Extract and NPs on the Migration of Primary Epidermal Keratinocytes, and HO-1-N-1 Cells

**Figure 7:**
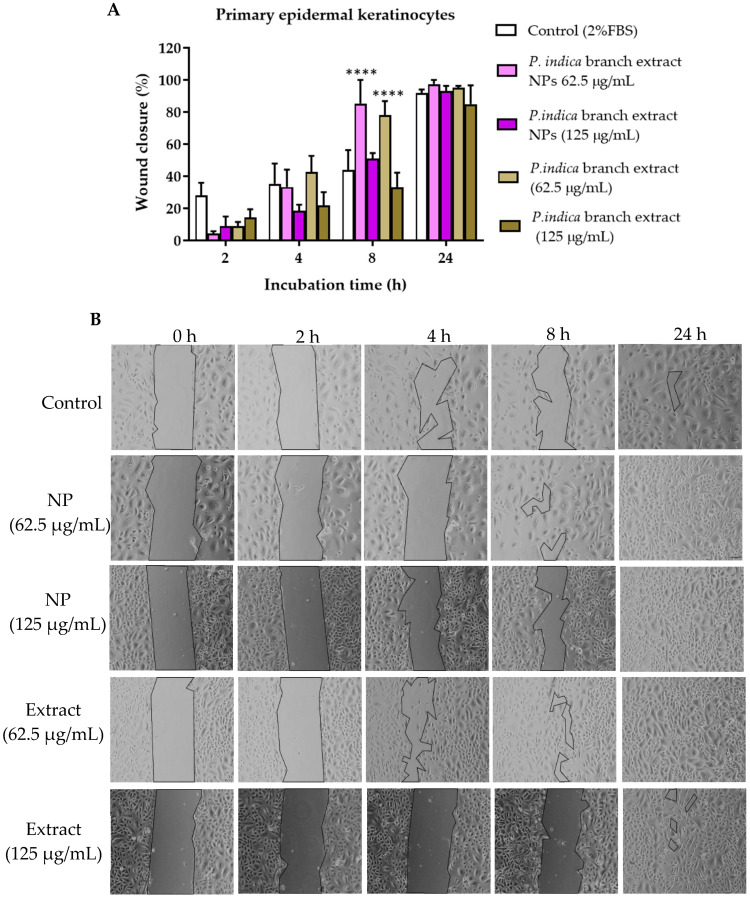
(**A**) Microscopic images of primary epidermal keratinocytes treated with *P. indica* branch extract in the scratch assay. (**B**) The images were captured at 0, 2, 4, 8, and 24 h after incubation. The data represent the mean ± SEM of three experiments. **** indicates *p* < 0.0001 compared with control.

**Figure 8:**
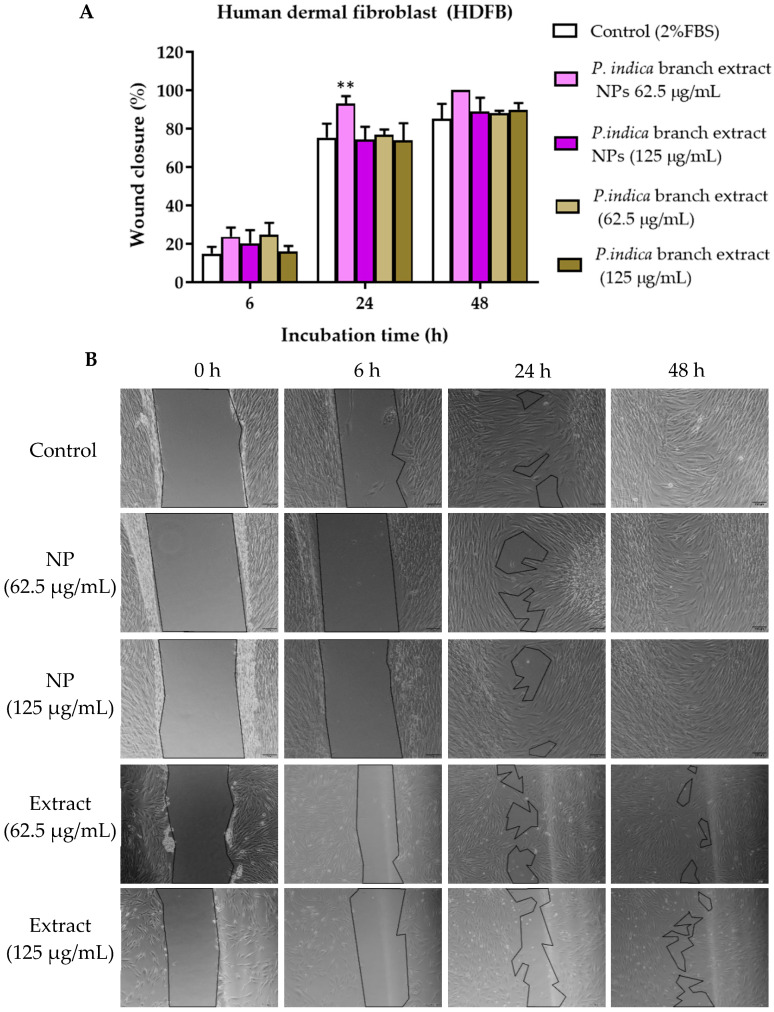
(**A**) Microscopic images of human dermal fibroblasts treated with *P. indica* branch extract in the scratch assay. (**B**) The images were captured at 0, 6, 24, and 48 h after incubation. The data represent the mean ± SEM of three experiments. ** indicates *p* < 0.01 compared with control.

**Figure 9:**
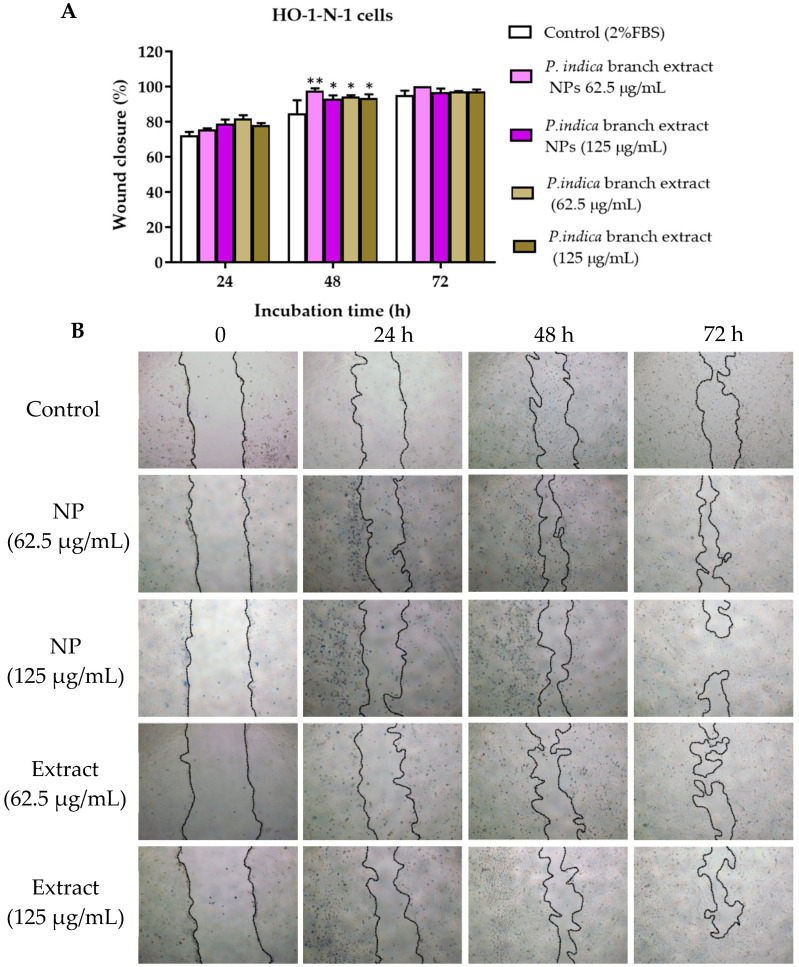
(**A**) Microscopic images of HO-1-N-1 cells treated with *P. indica* branch extract and NPs in the scratch assay. (**B**) The images were captured at 0, 24, 48, and 72 h after incubation. The data represent mean ± SEM of three experiments. * and ** indicate *p* < 0.05 and *p* < 0.01, respectively compared with control.

The scratch assay was conducted to observe cell migration, which plays an important role in wound healing. The concentrations of the extract and NPs that were not toxic to primary epidermal keratinocytes, human dermal fibroblasts and HO-1-N-1 cells, i.e., 62.5 and 125 µg/mL were chosen from the cell viability assays. After 2 h exposure to *P. indica* branch extract and NPs, all cells treated with extract and NPs and untreated cells migrated towards the gap. The wound closure percentage was calculated based on the reduction of the gap area compared with the initial gap area. The results showed that at 8 h after incubation, primary epidermal keratinocytes treated with 62.5 µg/mL of NPs and extract and showed a significant increase in migration with the % wound closure of 85.39 ± 14.61% and 78.09 ± 8.77%, respectively when compared with the control group (44.05 ± 12.28%) (Figure 7A,B). At 24 h after incubation, the migration rate of human dermal fibroblasts treated with 62.5 µg/mL of NPs was significantly higher than the control. The % wound closure of human dermal fibroblasts treated with 62.5 µg/mL of NPs and control were 93.03 ± 3.91% and 75.37 ± 7.28%, respectively (Figure 8A,B). The NPs and extract at 62.5 and 125 µg/mL significantly increased migration of HO-1-N-1 cells compared with the control. The micrographs of cells are shown in (Figure 9A,B).

## 4. Discussion

Wound healing consists of hemostasis, inflammation, proliferation, epithelization, and remodeling processes. Epithelization is the formation of an epithelial layer over the wound area, involving migration, differentiation, detaching, proliferation, and stratification of keratinocytes to form the new epithelium. Fibroblasts are common stromal cells present in human connective tissue. Fibroblasts play a critical role in wound healing by migration, proliferation, and secreting growth factors, cytokines, collagens, and extracellular matrix components to reconstitute the various connective tissue components [18].

Various plants used in traditional medicine have been investigated for wound healing properties. The wound healing activity of the crude plant extract results from plant secondary metabolites. In this study, phenolic acid, flavonoids, tannins, terpenoids, and alkaloids were detected in *P. indica* branch ethanolic extract. *P. indica* leaf extract has been demonstrated to contain flavonoids and phenolic compounds. Sugiaman et al. reported that there was 19.44 mg of flavonoids in 1 g of *P. indica* leaf ethanol extract [19]. Noridayu et al. revealed that *P. indica* leaf methanol extract had 5.74 mg per 1 g crude extract, whereas the hexane stem extract had 0.63 mg per 1 g crude extract [20]. Phenolic compounds, flavonoids, and tannins have been reported for wound healing due to their anti-microbial and antioxidant properties [21]. Flavonoids were known to reduce lipid peroxidation resulting in preventing cell damage and promoting DNA synthesis. Flavonoids have anti-inflammatory effects by inhibiting the cyclooxygenase enzyme. They also have antioxidant and antibacterial activities. Flavonoids have been shown to increase wound healing by accelerating the rate of epithelialization through the induction of the production of transforming growth factor-beta (TGF β). Flavonoids, tannins, and triterpenoids were also known to promote the wound healing process mainly due to their astringent and anti-microbial properties, responsible for wound contraction and accelerated epithelization. In addition, alkaloids are generally known to have anti-inflammatory, anti-microbial and wound healing activity [22]. 

The solvent displacement method is a simple and reproducible method for preparing nanoparticles. This method involves adding the organic phase consisting of *P. indica* crude extract dissolved in a water-miscible solvent (95% ethanol) with an aqueous phase consisting of sodium carboxymethyl cellulose or poloxamer as stabilizers. Nanoparticles were spontaneously formed in the continuous phase when the organic solution containing *P. indica* branch extract was added, resulting in a colloidal dispersion. Ethanol rapidly diffused out from the extract and accordingly formed nanometer-size nanoparticles, as a result of the Marangoni effect [23]. SCMC and poloxamer effectively produced acceptable size, polydispersity index and zeta potential of *P. indica* branch extract NPs. Our results showed that the size of 0.1% SCMC *P. indica* branch extract NPs was not significantly increased up until 4 month-storage, whereas the size of 0.1%poloxamer *P. indica* branch extract significantly increased within a week. These results indicated the greater stabilizing effect of SCMC compared with poloxamer at the same concentration. The higher stabilizing activity of SCMC was probably due to the different stabilizing mechanisms of the two polymers. Both poloxamer 407 and SCMC have been used to stabilize NPs [9,24]. Their stabilization mechanisms include steric stability and repulsive hydration force to the hydrophilic interface constructed by polymers on the surface of the NPs [25]. In addition, SCMC possesses a negative charge of carboxyl groups, providing repulsive electrostatic force which is also responsible for the stabilization of *P. indica* branch extract NPs. Poloxamer coated *P. indica* branch extract NPs had zeta potential values ranging from −3.8 to −7.3 mV. Compared with SCMC, the stabilizing mechanism of poloxamer which is a non-ionic surfactant was only dominated by steric hindrance and repulsive hydration forces with weak electrostatic repulsion [26]. Furthermore, the amount of adsorbed polymer on the NP surface also influences the colloidal stability of the NPs. These are dependent on polymeric structure, molecular weight, and adsorption energy [27,28]. Zeta potential is an important factor in characterizing the stability of colloidal dispersion. It was reported that a zeta potential negatively higher than −30 mV was required for electrostatic stabilization. The zeta potential values of 0.1% SCMC coated *P. indica* branch extract NPs ranged from −29.83 to −40.23 mV, which could keep the NPs stable. Per the optimal size, polydispersity index, and zeta potential values, NPs prepared using 0.1% SCMC were selected for further investigation.

The cytotoxicity of *P. indica* branch extract and NPs was indicated by the half-maximal inhibitory concentration (IC_50_) values. The IC_50_ values of NPs against primary epidermal keratinocytes and HO-1-N-1 cells were lower than that of the extract. The American National Cancer Institute (NCI) guidelines set the limit of activity for crude extracts at 50% inhibition (IC_50_) of the proliferation of less than 30 µg/mL after the exposure time of 72 h [29]. In this study, the IC_50_ values of the extract and NPs against primary epidermal keratinocytes and oral mucosal keratinocyte cells were much higher than the recommended thresholds suggesting the biocompatibility of the extract and NPs with these cell lines. The percentage viability of fibroblasts was not affected after exposure to extract and NPs.

The migration and proliferation of keratinocytes, dermal fibroblasts, and oral mucosal cells are pivotal to the epithelization process of wound healing. In this study, the migration rates of primary epidermal keratinocytes, human dermal fibroblasts, and oral mucosal cells after being treated with *P. indica* branch extract and NPs were assessed by the in vitro scratch assay. A percentage of the closed area was measured after the determined incubation period and compared with the control. An increase in the percentage of wound closure indicated cell migration. The results suggested that a low concentration of *P. indica* branch extract NPs (62.5 µg/mL) accelerated wound healing by increasing the migratory rates of all cell types. The increase in cell migration rate of *P. indica* branch extract might be attributed to the secondary metabolites in the extract, including phenolic acid, flavonoids, tannins, and terpenoids individually or with additive effect [10].

4, 5-*O*-Dicaffeoylquinic acid is a polyphenol compound that has been detected as one of the bioactive compounds in *P. indica* (L.) Less leaves [30,31,32]. We have previously reported that *P. indica* (L.) Less leaves demonstrated in vitro wound healing properties in a time and dose-dependent manner [9]. 4, 5-*O*-Dicaffeoylquinic acid was identified in plants possessing antioxidant and wound healing activities. Kamarauskaite et al. revealed the antioxidant activity of caffeoylquinic acid-rich fractions from Artemisia species herb extracts based on several mechanisms of action [33]. The aerial part of *Scorzonera* spp. Including *Scorzonera baetica (Boiss.) Boiss.*, *Scorzonera crispatula Boiss*., *Scorzonera hispanica L.*, and *Scorzonera baetica (Boiss.) Boiss.* contained caffeoylquinic acid derivatives, including 4,5-*O*-dicaffeoylquinic acid [34]. These plant species have potential antioxidant, anti-inflammatory, pain-relieving, and wound healing properties. Free radicals attack important macromolecules, such as membrane lipids, enzymes, and nucleic acids, leading to damage to cells and tissue [35]. Therefore, the free radical scavenging activities of *P. indica* branch extract and NPs might prevent keratinocytes and fibroblasts from cell and tissue damage and promote wound healing. In this study, we firstly reported that 4,5-*O*-dicaffeoylquinic acid was a major compound detected in *P. indica* (L.) Less branch ethanol extract. This bioactive compound was hypothesized to possess migration-enhancing effects on keratinocytes and fibroblasts in vitro wound healing studies.

## 5. Conclusions

Phytochemical screening revealed that *P. indica* branch ethanol extract presented phenolic acid, flavonoids, tannins, alkaloids, and terpenes. The validated HPLC analysis revealed that the major bioactive compound in *P. indica* branch ethanol extract was 4,5-*O*-dicaffeoylquinic acid. *P. indica* branch extract and NPs did not affect the viability of human dermal fibroblasts. The toxicity of *P. indica* branch extract and NPs against primary epidermal keratinocytes and oral mucosal keratinocyte cells was dose-dependent. *P. indica* branch extract and NPs at specified concentrations accelerated the skin/oral mucosal cell migration in comparison with natural cell migration, probably due to antioxidant activity.

## Figures and Tables

**Table 1:** IC_50_ values of *P. indica* branch extract and NPs.

Cell Type	*P. indica* Branch Extract (µg/mL)	*P. indica* Branch Extract NPs (µg/mL)
Primary human keratinocytes		
24 h	453.6 ± 6.9	342.2 ± 27.7
Human dermal fibroblasts		
24 h	N/A	N/A
48 h	N/A	N/A
72 h	N/A	N/A
HO-1-N-1 cells		
24 h	798.0 ± 7.8	698.4 ± 3.7
48 h	778.6 ± 6.4	543.2 ± 3.2
72 h	N/A	N/A

## Sample Availability

Samples of the compounds are not available from the authors.
